# Asthma in an Urban Population in Portugal: A prevalence study

**DOI:** 10.1186/1471-2458-11-347

**Published:** 2011-05-19

**Authors:** Jaime Correia de Sousa, Maria Espírito Santo, Tânia Colaço, Filipa Almada-Lobo, John Yaphe

**Affiliations:** 1 Life and Health Sciences Research Institute (ICVS), School of Health Sciences, University of Minho, Portugal and Horizonte Family Health Unit, Matosinhos, Porto, Portugal; 2 Horizonte Family Health Unit, Matosinhos, Porto, Portugal; 3 Porta do Sol Family Health Unit, Matosinhos, Porto, Portugal; 4 Life and Health Sciences Research Institute (ICVS), School of Health Sciences, University of Minho, Portugal

**Keywords:** Asthma, Prevalence, Portugal

## Abstract

**Background:**

The prevalence and incidence of asthma are believed to be increasing but research on the true incidence, prevalence and mortality from asthma has met methodological obstacles since it has been difficult to define and diagnose asthma in epidemiological terms. New and widely accepted diagnostic criteria for asthma present opportunities for progress in this field. Studies conducted in Portugal have estimated the disease prevalence between 3% and 15%. Available epidemiological data present a significant variability due to methodological obstacles.

**Aim:**

To estimate the true prevalence of asthma by gender and age groups in the population of the area covered by one urban Health Centre in Portugal.

**Method:**

An observational study was conducted between February and July 2009 at the Horizonte Family Health Unit in Matosinhos, Portugal. A random sample of 590 patients, stratified by age and gender was obtained from the practice database of registered patients. Data was collected using a patient questionnaire based on respiratory symptoms and the physician's best knowledge of the patient's asthma status. The prevalence of asthma was calculated by age and gender.

**Results:**

Data were obtained from 576 patients (97.6% response rate). The mean age for patients with asthma was 27.0 years (95% CI: 20.95 to 33.16). This was lower than the mean age for non-asthmatics but the difference was not statistically significant. Asthma was diagnosed in 59 persons giving a prevalence of 10.24% (95% CI: 8.16 to 12.32). There was no statistically significant difference in the prevalence of asthma by gender.

**Conclusion:**

The prevalence of asthma found in the present study was higher than that found in some studies, though lower than that found in other studies. Further studies in other regions of Portugal are required to confirm these findings.

## Background

Asthma is a common chronic disease world-wide with a high prevalence in all age groups, mostly in children and young people yet there is wide variation in the prevalence of the condition between and within countries [1-8]. Asthma prevalence and incidence are believed to be increasing [9-11], though this view is controversial [2,12,13].

Asthma is also an important reason for hospital admission [14,15] and causes considerable limitations on the physical, emotional, social, and professional lives of both patients and their families, interfering with normal activity and quality of life [16,17].

Available epidemiological data on the incidence, prevalence and mortality from asthma present significant variability as research has met with methodological obstacles since it is difficult to define and diagnose asthma in epidemiological terms [2,18,19]. Some studies used non-standardized questionnaires while other included patients after a pulmonary function assessment or proof of bronchial responsiveness. Because of the differences in methodology, considerable differences of prevalence have been found in different studies. New widely accepted diagnostic criteria for asthma present opportunities for progress in this field.

The Global Initiative for Asthma (GINA) lists some actions required to reduce the burden of asthma. One task is to recognise asthma as an important cause of morbidity, economic cost, and mortality worldwide and to measure and monitor the prevalence of asthma, and the morbidity and mortality due to asthma throughout the world [2].

The Portuguese Asthma Control Program was developed in 2000 [20] and, in line with the GINA proposal, recommended regular studies on epidemiological and clinical follow up of asthma. The Portuguese Guidelines on the diagnostic and control of asthma, based on the existing GINA Guidelines, were also published in 2000 [21] and were updated in 2007 [22] after the 2006 changes of the GINA Report.

Several studies conducted in Portugal have estimated the disease prevalence as ranging from 3.3% to 15% [23-32]. Most of the Portuguese data available were obtained before 2006, covered limited age groups or were obtained using methodologies with questionable accuracy of the prevalence rates.

This study was conducted to determine the prevalence of asthma by gender and stratified age groups in the Horizonte Family Health Unit in Matosinhos Portugal in 2009 to increase our knowledge of the epidemiology of asthma.

## Method

### Study population

Matosinhos, Portugal is a suburb of the city of Porto with a population of 169,261 inhabitants in 2008. The age and gender distribution of Matosinhos is similar to that of the total Portuguese population [33]. The population of the *Horizonte *Family Health Unit is similar in age and gender distribution in turn to the population of Portugal (Additional file 1, Appendix 1 - Comparison of the practice population with the Portuguese Population).

The *Horizonte *Family Health Unit is a group practice formed by eight family physicians working together for more than ten years with a stable registered population. The change in the population of registered patients in the practice between 2008 and 2009 was an increase of 1.88%. There is a good accessibility to healthcare. Most patients can be seen by any of the partners in case of their personal doctor is absent with continuity of the electronic medical record. The unit is a training practice for basic medical education and postgraduate family medicine residency training programmes. These characteristics of the practice provide a unique opportunity for epidemiological studies.

An observational study was conducted between February and July 2009 at the Horizonte Family Health Unit. A stratified random sample by age and gender was obtained from the practice general database of 13,568 registered patients.

### Justification of age strata

The study population was divided into four age strata: 0 to 7 years, 8 to 19 years, 20 to 64 years and over 65 years. The youngest age group of birth to 7 years was selected to represent the age when asthma tends to be most prevalent and hospital admission rates are higher. The second age group of 8 to 19 years was chosen to study the period when mortality from asthma has been found to be highest [34]. Adults aged 20 to 64 and the elderly aged 65 and over were studied separately because diagnostic problems regarding asthma are common in the elderly.

### Sampling procedures

Based on the available published literature a prevalence of asthma was predicted in this population for the four age strata. In the 0 to 7 year age group a prevalence of 11% was predicted, for 8-19 years 11.8%, for 20 to 64 years 5%, and for over 65 years 5%. Using the method of Lehr [35], a sample size calculation was performed to determine the true prevalence of asthma in each age group plus or minus 2% with 95% confidence. In the 0 to 7 year age group 150 patients would be required, for 8-19 years 160 patients, for 20 to 64 years 140 patients, and for over 65 years 140 patients. Thus a sample of 590 patients would be required in this population.

The required number of patients was selected from each age group using the patient register in the practice and a random number generator.

## Data collection

Data were collected using two questionnaires: a physician's questionnaire (Additional file 2, Appendix 2 - Physician Questionnaire) and a patient questionnaire (Additional file 3, Appendix 3 - Patient Questionnaire). The physician's questionnaire consisted of demographic information including the age and gender of each of the selected patients and clinical data on asthma. The first question was "does this patient have asthma?" If the answer was positive, the family doctor was required to answer four sets of questions. The first questions asked about the presence of wheezing. The second set assessed a past or present history of dry cough, recurrent wheeze, dyspnoea/recurrent difficulty in breathing or recurrent chest tightness. The third set of questions assessed the evidence of reversibility of airflow obstruction after administration of a short acting bronchodilator through clinical observation only, peak flow measurement or spirometry. The fourth question asked if a diagnosis of asthma had been made by a secondary care specialist in case the diagnosis had not been made by the family doctor alone.

**Figure 1:**
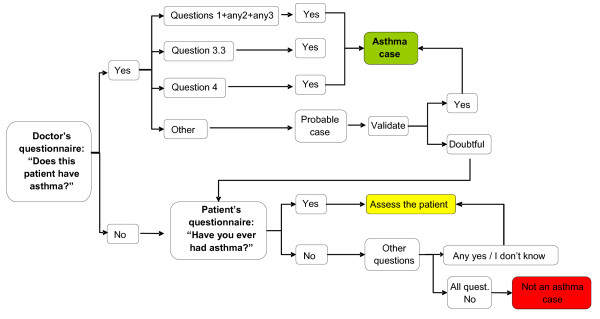
Steps to the diagnosis or exclusion of asthma in the asthma prevalence study

The patients' questionnaire was based on the ISAAC questionnaire [36] and consisted of demographic information including age and gender and 13 questions on asthma. Eight questions where about symptoms and used the ISAAC core questionnaire for wheezing and asthma. The first group of questions asked about the presence of wheezing at present and specifically in the last 12 months, the number of attacks of wheezing in the last 12 months, wheezing during or after exercising, and waking up because of wheezing or dry cough at night in the last 12 months. Patients were asked if they ever had asthma or if they had ever been told by a doctor that they had asthma. The second section consisted on five questions about medication. Questions were asked on the use of any form of asthma medication in the last 12 months and covered all the commercial alternatives available in Portugal including the colour and the brand names of both inhalers and tablets. An algorithm of diagnostic steps was used to make the diagnosis of asthma based on the responses obtained in the study questionnaires (Figure 1).

In the first phase of the study, between February and April 2009, all eight physicians in the health unit were asked to report on each of the selected patients that were seen during routine scheduled appointments for any clinical reason. Each family doctor completed the physician's questionnaire. Each patient was invited to answer a questionnaire based on respiratory symptoms and signed an informed consent form.

In the second phase, between April and July 2009, all the patients who had missed an appointment were contacted by phone and invited for an interview with the researchers, while the family physician was asked to fill in the doctor's questionnaire. Any patient who failed to answer the phone after three attempts or refused to participate was replaced by another patient with similar age and gender characteristics (next random number in the same age group). Patients who did not have any appointment during the first phase were also contacted by phone.

The diagnostic procedures were based on a combination of the answers given by the patient on respiratory symptoms and the physician's best knowledge of the patient's asthma status. If the family doctor answered "no" to the question "Does this patient have asthma?" and the patient reported the absence of asthma symptoms, it was considered that asthma was not present. If the doctor answered "yes" to the same question and the patient reported symptoms of asthma ("probable cases"), the diagnostic algorithm was used (figure 1) in order to confirm or reject the diagnosis. In cases of inconsistency between the information provided by the family doctor and the patient ("doubtful cases"), an interview was conducted by the researchers with an assessment of the patient to validate the diagnosis.

### Data analysis

Data were entered on an electronic spreadsheet program and analyzed using Epi-Info version 3.5.1 software. The prevalence of asthma was calculated for the total population and for specific age groups by dividing the number of cases found by the size of the population. Standardized rates were computed using Portuguese census data for 2005 [33].

In order to assess the predictive value of the items used in the symptom questionnaire and the diagnostic algorithm described above for a diagnosis of probable asthma or doubtful asthma, odds ratios and 95% confidence intervals were calculated using the Epi-Info TABLES function. This was done to determine which items in the history were most likely to be associated with a final diagnosis of asthma.

### Ethical approval

Prior to data collection, the study protocol was approved by the Ethics Committee of the Local Health Authority in Matosinhos (Unidade Local de Saúde de Matosinhos). Each patient invited to participate signed an informed consent form after the aims and methods of the study were explained. All new cases of asthma or uncontrolled cases found in the study were referred back to their family physician for treatment.

## Results

**Figure 2:**
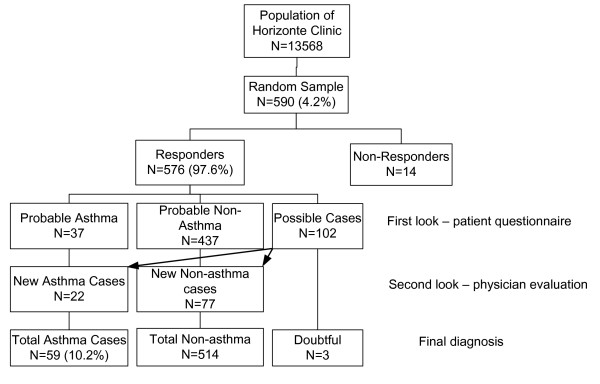
Flow chart of patient recruitment and evaluation in asthma prevalence study

From the stratified random sample of 590 patients selected from the population of the Horizonte Family Health Unit, data were obtained from 576 patients (for a response rate of 97.6%, Figure 2). The characteristics of the study population are given in Table 1.

**Table 1:** Demographic characteristics of study sample by gender, age and diagnosis

	Total sample N = 576 Mean age	Asthma cases N = 59 Mean age	Non-asthma N = 517 Mean age
	33.2 years (s.d. 28.1)	27.0 years (s.d. 23.4)	33.9 years (s.d. 28.5)

Males	32.2 years (s.d. 27.1) N = 279 (48.4%)	22.0 years (s.d. 17.8) N = 29	33.4 years (s.d. 27.7) N = 250

Females	34.2 years (s.d. 28.9) N = 297 (51.6%)	31.8 years (s.d. 27.2) N = 30	34.5 years (s.d.29.2) N = 267

The mean age of this population was 33.2 years (sd = 28.1 years; 95% CI: 30.9 to 35.5), and 297 (51.6%) of the patients in the sample were female.

Using the GINA criteria, asthma was diagnosed in 59 persons giving a prevalence of 10.24% (95% CI: 7.76 to 12.72).

A diagnosis of "probable asthma" was initially made in 37 patients (6.4%) and 102 patients were classified as "doubtful cases"

Following a medical record review and interview of doubtful cases, 22 were reclassified as asthmatic (for a total of 59 cases), 514 as healthy and 3 (0.5%) remained as doubtful cases.

The mean age for patients with asthma was 27.0 years (sd 23.4, 95% CI: 21.0 to 32.9). For non-asthmatics the mean age was 33.9 years (sd 28.5, 95% CI: 31.4 to 36.3). This difference was not statistically significant.

The mean age for male patients with asthma was 22.0 years (sd 17.8; 95% CI: 15.5 to 28.5) with a median age of 18.3 years. For males without asthma the mean age was 33.4 (sd 27.7; 95% CI: 29 to 36) with a median age of 19.7 years. This difference was statistically significant (p < 0.05).

The mean age for female patients with asthma was 34.2 years (sd 28.9; 95% CI: to 41.5) with a median age of 18.2 years. For females without asthma the mean age was 34.5 (sd 29.2; 95% CI: 31 to 38) with a median of 19.3 years. This difference was not statistically significant.

Female patients comprised 51.8% of the asthma patients. There was no statistically significant difference in the prevalence of asthma by gender for the total population.

Among young children under the age of 8 years the prevalence of asthma is higher in males (13%) than in females (7%) but this difference is not statistically significant in this population (OR = 2, 95% CI, 0.6 to 6.9).

Among patients over 65 years of age the prevalence is higher in females (7.3%) than in males (1.7%) but this difference is not statistically significant (OR = 0.2, 95% CI = 0.004 to 1.9).

Asthma prevalence by age group in the sample population and standardized for the Portuguese population is given in table 2.

**Table 2:** Asthma prevalence by age group in the sample population and standardized for the Portuguese population

Age groups	Asthma	Non-asthma	Prevalence %	95% CI	Prevalence % Standardized for Portuguese Population
0 to 7	13	123	9.56	5.41 to 13.71	8.6

8 to 19	21	139	13.13	8.74 to 17.52	13.1

20 to 64	18	122	12.86	8.21 to 17.51	12.3

≥ 65	7	133	5.00	1.97 to 8.03	6.3

Total	59	517	10.24	8.16 to 12.32	

Total sample		576			

(The three "doubtful cases" were included with the non-asthma patients in this table.)

### Associations between symptoms and diagnosis

The association between positive symptoms elicited in the patient questionnaire and a final diagnosis of asthma was tested by computing the odds ratio (OR) for a positive reply comparing true cases of asthma with doubtful cases (Table 3). The following items were found to be statistically significant: wheezing after exercise (OR 4.2), waking up from wheezing (OR 3.4), a personal history of asthma (OR 12), being told by a doctor that the patient has asthma (OR 14.6), use of a short-acting beta agonist in last 12 months, (OR 35.6), use of a long-acting beta agonist in last 12 months (OR 7.6), and the use of an inhaled steroid in last 12 months (OR 25).

**Table 3:** Odds ratios for the association of positive symptoms with a diagnosis of asthma

**From the patients' questionnaire, the following items were positive**:	Asthma cases (n = 59)	Doubtful cases (n = 39)	O.R. (95% CI)
Any history of wheezing	57 (96%)	38 (97%)	

Wheezing in the last 12 months	40 (67%)	22 (26%)	

Wheezing after exercise	19 (32%)	4 (10%)	4.2 (1.2-18.1)

Wake up from wheezing	23 (39%)	6 (15%)	3.4 (1.1-11.7)

Night time dry cough	40 (67%)	20 (51%)	

Personal history of asthma	45 (76%)	8 (20%)	12 (4.2-37.9)

Told by a doctor they have asthma	47 (80%)	8 (20%)	14.6 (5-47)

Use of SABA in last 12 months	29 (49%)	1 (3%)	35.6 (5-1358)

Use of LABA in last 12 months	10 (17%)	1 (3%)	7.6 (1.00-345)

Use of Inhaled steroid in last 12 months	24 (40%)	1 (3%)	25 (3.7-1099)

Use of LABA-steroid in last 12 months	4 (7%)	1 (3%)	

Use of leukotriene in last 12 months	11 (19%)	0	

## Discussion

This study found that the prevalence of asthma in the population of the area covered by the Horizonte Family Health Unit in Matosinhos, Portugal was 10.24% using data collected from a random sample of the registered population.

In Portugal, the composition of the population of patients registered on the lists of general practitioners is very similar to the general population living in the region. This was described in a recent publication on the incidence of asthma using general practice populations [37]. The population described in the general practice in current study is believed to be representative of Matosinhos therefore these findings are believed to represent the prevalence of asthma in this population.

### Internal validity

The findings of this study are believed to represent the true prevalence of asthma in this population. This is due to use of a stratified random sample drawn from accurate patient lists with a good response rate. In the case of non-responders, replacements were drawn from a list of alternates also produced by the random number generator. The use of accepted diagnostic criteria validated by additional interview and examination by physicians in cases of doubt regarding the diagnosis also increases confidence in the quality of the data. In some age groups the wide confidence intervals for the prevalence of asthma is due to the small numbers of cases found. A similar study in a larger population will resolve this issue further.

### Study questionnaire and diagnostic criteria

The patient questionnaire was based on the Portuguese version of the ISAAC questionnaire which had been previously used in Portugal as part of an international study [4]. (The authors used only the relevant questions about asthma symptoms from the questionnaire.)

The physician questionnaire was constructed using the guidelines in the GINA 2006 Report [38]. Questions were asked about the presence of wheezing, past or present history of dry cough, recurrent wheeze, dyspnoea/recurrent difficulty in breathing or recurrent chest tightness and the evidence of reversibility of airflow obstruction after administration of a short acting bronchodilator through clinical observation only, peak flow measurement or spirometry. The same questionnaire was used in a study on the incidence of asthma and accuracy of diagnosis in the Portuguese sentinel practice network [37]. The utility of this questionnaire in these two studies suggests that future study of asthma prevalence could also use these diagnostic criteria.

### Comparison of findings with other published studies

There are many published studies on the prevalence of asthma but the absence of a precise and universally accepted definition of asthma makes reliable comparison of reported prevalence from different parts of the world problematic [2].

### Portuguese studies

The prevalence of asthma found in the present study for the general population was considerably higher than that found in previous studies in Portugal though different methodologies and age groups have been used. Care is required in comparison of these results with findings from other studies. In the survey published in 1987 by Nunes et al [24] for patients aged 7 to 17 years observed regularly using a symptom score and spirometry, a prevalence of 3.4% was found. In 1990 Chieira et al [25] studied 557 twenty year old male conscripts using a questionnaire and clinical observation and found a prevalence of 5.2%. In 1991 Marques [39] studied a random sample of 2000 inhabitants of the city of Porto aged 20 to 44 years using the ECRHS questionnaire and found a prevalence of 4.3%. In 1992 Nunes et al [26] published a report on 55,254 subjects in Algarve, Portugal using a questionnaire and found a prevalence of 5.5% among patients attending primary care consultations. In 1994 Alves et al [23] published the results of a survey in the city of Porto using a postal questionnaire and found a prevalence of 3.1% for the diagnosis of asthma and 6.45% for asthma symptoms in persons of both genders aged 18 to 44 years. In 1994 Prata et al [27] studied 927 children 6-12 yrs old in Faial, Azores, and met a prevalence of 8.0%. In 1995 Vicente et al [28] studied 17,200 students aged 12-19 yrs living in eight major cities in Portugal and found a prevalence of 3.2%. In 1996 Morais Almeida et al [29] used a questionnaire to study a sample of 1,061 children aged 6-10 yrs in the Madeira Island, Portugal and met a prevalence of 15%. In 1998 Leiria Pinto P et al [30] studied 1,334 adolescents aged 12-16 yrs living in Lisbon using a questionnaire and found a prevalence of 11.4%.

The 2005 Portuguese National Health Survey [40] found an overall asthma prevalence of 5.5%. The method used for data collection was a home interview of a stratified representative sample of the Portuguese population with self-report on the conditions of interest. In 2005 Branco et al [31] used a telephone interview with a random sample of 1,211 households in mainland Portugal and a total of 2,820 subjects of all ages as part of a survey of chronic conditions. The prevalence of self-reported asthma was 8.6%. Table 4 displays a comparison of asthma prevalence rates for all age groups and methods in Portuguese surveys.

**Table 4:** Comparison of asthma prevalence rates for all age groups and methods in Portuguese surveys

Year	Study	Prevalence %	Method
1992	Nunes et al [26]	5.5	Questionnaire

2005	Branco et al [31]	8.6	Self-report/Interview

2006	Portuguese National Health Survey [40]	5.5	Self-report/Interview

2009	Correia-de-Sousa et al	10.2	Questionnaire, self-report of symptoms

### International studies

The comparison of the Matosinhos survey results with other international data confirms that they are close to published values. According to data from the World Health Survey from 2003, [41] the prevalence of diagnosed asthma showed a narrow range across countries. There was a 10-fold variation in current wheezing symptom prevalence across countries (2.4% in Vietnam and 24.3% in Brazil). The majority of estimates fell within a fourfold range of prevalence, with Vietnam the lowest at 1.8% and Australia the highest at 32.8%.

The 2003 Global Burden of Asthma document [3] published on behalf of the GINA project compares the mean prevalence of clinical asthma in different regions. It ranges from 2.1% in Central North Asia (China/Taiwan/Mongolia) to 16.1% in the United Kingdom and Republic of Ireland. Prevalences are higher in North America, Oceania and West and Eastern Europe than in Asia or Africa. Southern Africa (8.1%) and South America (9.9%) have intermediate prevalences. The prevalence given for Western Europe (including Portugal) is 5.9%, lower that the data from the Matosinhos study. These studies have gathered data from different sources and present a rather different range of values.

The most recent publications suggest that asthma prevalence ranges from 7.2% to 12.2%. In the U.S. National Surveillance for Asthma study published in 2007 the overall prevalence of asthma was 7.2% [42]. A Germany study from 2005 by Stock et al [43] shows a total prevalence of asthma of 6.34% in the study population. A study from South Australia from 2006 by Wilson et al [44] shows that asthma prevalence increased from 7.5% to 12.2% from 1990 to 2003. In a Swedish study from 2009 by Lötvall et al [45], asthma prevalence, defined as asthma diagnosed by a physician, was 8.3%. In a Norwegian study from 2003 by Brogger et al [46], the crude prevalence of ever having had a doctor's diagnosis of asthma increased from 3.4 to 9.3% from 1972 to 1998-99. Table 5 presents a comparison of published international asthma prevalence rates.

**Table 5:** International comparison of asthma prevalence rates for all age groups

Year	Study	Country	Prevalence %
2003	Brogger et al [46]	Norway	9.3

2005	Stcok et al [43]	Germany	6.34

2006	Wilson et al [44]	Australia	12.2

2007	National Surveillance for Asthma US [42]	USA	7.2

2009	Lötvall et al [45]	Sweden	8.3

2009	Correia-de-Sousa et al	Portugal	10.2

### Comparison of prevalence by age groups

In the United States National Surveillance for Asthma, the prevalence rate among the birth to 4 year age group was 5.9% [42]. A study among kindergartners in Chicago public schools (mean age 5.7 yrs) found a prevalence of asthma of 10.8% [47]. Data published by The Los Angeles County Health Survey [48] shows a prevalence of asthma in the birth to 5 years group of 5.9%. Martinez et al. [49] studied wheezing before the age of three years and the relation to wheezing at six years of age and found that at the age of six years 13.7% of the studied children had wheezing both before three and at six years of age. Children with persistent wheezing had significantly reduced lung function. According to data from the New York State Asthma Surveillance, children from birth to 4 years in New York State had an asthma prevalence of 7.5% in 2006-2008 [50]. In a study carried out in Asturias, Spain, asthma prevalence calculated for infants was 7.6% [51]. In the Matosinhos survey, asthma prevalence in the birth to 7 years age group was 9.59%, close to figures found in published data.

In the study from 2008 by Ramos et al [32] in a sample of 13 year old urban adolescents the lifetime prevalence of asthma was 12.9%, with 84.4% of the diagnoses confirmed by a physician. In the Matosinhos survey, the prevalence in the age group 8 to 19 years was 13.13%. In the study by Nunes [24] the prevalence for a similar age group (7 to 17 years) was 3.4%. Vicente et al [28] found a prevalence of 3.2% in those aged 12 to 19 years old. In the age group 20 to 64 years, our study showed a prevalence of 12.86% while other Portuguese surveys found a prevalence of 3.1% and 4.3% [23,39]. There were no Portuguese data found for comparison of either the lower age groups (0 to 7 years) or for the elderly (over 65 years).

A Swedish study from 2006 [52] in children aged 7 to 8 years reported an increase in prevalence from 1996 to 2006 from 5.7% to 7.4%. In New Zealand the prevalence of asthma is considerably higher. In the ISAAC Phase Three study by Asher et al [53] repeating the questionnaire survey of two age groups of school children (6 to7 years and 13 to 14 years), the prevalence rates found were 30.2% and 32.4% respectively. The results of Phase III of the International Study of Asthma and Allergies in Childhood (ISAAC) [12] in the age groups 6 to7 years and 13 to 14 years provide data for international comparison of prevalence rates for asthma and wheezing. The Portuguese data from this study show a mean lifetime prevalence of asthma in the age group 6 to7 years of 9.8% and 13.2% for the 13 to 14 year age group. These results are similar to our findings. The U.S. National Surveillance for Asthma study [42] found the prevalence to be 8.5% among those under 18 years and 6-7% for those over 18.

The U.S. National Health and Nutrition Examination Survey (NHANES III) [54] found an asthma prevalence on the age group over 20 years of 4.5%. In the Danish survey by Browatzki et al [55] published in 2009, the prevalence of self-reported asthma in the age group 20 to 35 years was 6.9%.

In the U.S. National Surveillance for Asthma study [42] the lifetime prevalence of asthma in individuals over 65 years of age was found to be 6.8%, though the percentage of elderly patients with "current" asthma was 5.9%. In the NHANHES III survey [54], the prevalence over 60 years was 3.6%. In our study, the prevalence for the over 65 age group was 5%.

The comparison of the findings of the current study with other studies computing lifetime prevalence of asthma is relevant because the current study included questions assessing if the patent had ever been told they had asthma.

### Comparison of asthma prevalence by gender

Some studies report a clear gender difference [52,56], considering male gender to be associated with the increased risk of asthma in children. Prior to the age of 14, the prevalence of asthma may be nearly twice as great in boys as in girls [2].

In the present study there was no statistically significant difference in the prevalence of asthma by gender, though, among young children under the age of 8 years the prevalence of asthma is higher in males (13%) than in females (7%) but this was not statistically significant. Branco et al [31] also found no significant gender differences in asthma prevalence. This study was most similar to the Matosinhos survey. In some Portuguese surveys the overall prevalence for females is higher than for males [39,40], while in other the reverse was observed [32].

The U.S. National Surveillance for Asthma study [42] shows a difference in prevalence by genders with 8.1% among females and 6.2% among males. The report from the ISAAC phase I and III studies in Spain [56] shows a difference in prevalence by gender. In the age group 6 to 7 years, girls had 9.0% and boys 12.9%. In the age group 13 to 14 years, girls had 11.8% and boys 13.8%.

The Danish study of the prevalence and severity of asthma in young Danish adults over three decades shows a higher female prevalence in all the three phases. The female to male ratio was 1.7/1.2 in 1978, 4.8/4.5 in 1994 and 7.7/5.9 in 2004 [55].

## Discussion of methods

Using the best knowledge about asthma from both the patient and the physician was also used in a Swedish study published in 2005. The authors concluded that a combination of a medically verified asthma diagnosis using medical records and the use of self-reported symptoms with the ISAAC questionnaire seem to be valid and reliable for the follow-up childhood asthma in the community [57]. This is the method we chose for diagnosis in our study and it seems to be a feasible method for increasing diagnostic accuracy. It may explain the higher prevalence found in our study compared to other Portuguese studies.

### Implications

The findings of the current study suggest that asthma might have been under-reported in other epidemiological studies in Portugal. This may also be true in other countries. The use of accepted diagnostic criteria using the best knowledge from both the doctor and the patient may help to overcome this.

Careful assessment of symptoms can lead to more accurate diagnosis of population. Skilful application of this method in clinical practice may help to identify those patients who may benefit from appropriate treatment.

## Conclusions

Use of the best knowledge about asthma from both the patient and the physician seems to be a good strategy for the determination of the prevalence of asthma in a community survey.

The prevalence of asthma found in the present study was higher than that found in some studies, though other studies have found a prevalence above 10%.

The prevalence of asthma in the birth to 7 age group was lower than expected due to diagnostic problems.

Based on these data, assuming a prevalence of asthma of 10%, with the current population of Portugal of 10,893,010 in 2011, we may expect 1,089,301 persons to have asthma in Portugal.

Further studies in other regions of Portugal using the same diagnostic criteria and sampling methods should be done in order to confirm these findings.

## List of abbreviations used

GINA: Global Initiative for Asthma; ISAAC: International Study of Asthma and Allergies in Childhood; OR: Odds ratio; CI: Confidence interval;

## Competing interests

No conflicts of interests are reported for this study.

Dr. Correia de Sousa is an unpaid member of the scientific board of AstraZeneca Foundation Portugal. His department has received research funding from AstraZeneca in the past.

## Authors' contributions

JCS conceived the idea for the study, designed it, collected data and wrote parts of the introduction, methods, results and discussion sections of the manuscript. ME, TC and FAL participated in the design, collected data and contributed to the writing of the methods and results section. JY performed the data analysis and wrote parts of the introduction, methods results and discussion. All authors read and approved the final version of the manuscript.

## Pre-publication history

The pre-publication history for this paper can be accessed here:

http://www.biomedcentral.com/1471-2458/11/347/prepub

## Supplementary Material

Additional file 1 Appendix 1: Comparison of the practice population with the Portuguese Population Click here for file

Additional file 2 Appendix 2: Physician Questionnaire Click here for file

Additional file 3 Appendix 3: Patient Questionnaire Click here for file
