# Supplementary material for: Asthma in an Urban Population in Portugal: A prevalence study
Source: BMC Public Health. 2011 May 19;11:347. doi: 10.1186/1471-2458-11-347 (PMC3121634; doi:10.1186/1471-2458-11-347)
Supplement: Additional file 1 — Appendix 1: Comparison of the practice population with the Portuguese Population [file 1471-2458-11-347-S1.DOC]

Appendix 1: Comparison of the practice population with the Portuguese Population

|  | **Portugal** | | **Horizonte Clinic** | |
| --- | --- | --- | --- | --- |
| Age group | n | % | n | % |
| 0 to 7 years | 853,404 | 8.02 | 1,209 | 8.91 |
| 8 to 19 years | 1,329,915 | 12.50 | 1,706 | 12.57 |
| 20 to 64 years | 6,553,241 | 61.60 | 8,731 | 64.35 |
| ≥65 years | 1,901,153 | 17.87 | 1,922 | 14.17 |
| Total | 10,637,713 | 100.00 | 13,568 | 100.00 |
