# Supplementary material for: Asthma in an Urban Population in Portugal: A prevalence study
Source: BMC Public Health. 2011 May 19;11:347. doi: 10.1186/1471-2458-11-347 (PMC3121634; doi:10.1186/1471-2458-11-347)
Supplement: Additional file 2 — Appendix 2: Physician Questionnaire [file 1471-2458-11-347-S2.DOC]

**AppEndix 2: Physician questionnaire**

## Asthma prevalence study in Matosinhos Health Centre

| **Physician** | |_|_|_|_| (code) | **Date** (day/month/year) | __/__/_____ |
| --- | --- | --- | --- |

## Patient’s data

| **Age** | |_|_| years | **Gender |M|F|** | **Code** | ____________ | | | | |
| --- | --- | --- | --- | --- | --- | --- | --- | --- |
| **Does this patient have asthma?** | | | | | Y | N | I |  |

## If this patient has asthma mark down the criteria that supported the diagnosis

|  |  | | Y | N | I |
| --- | --- | --- | --- | --- | --- |
| **1.** | Wheezing | | 1 | 2 | 9 |
| **2.** | Past or present history of one of the following symptoms: | |  |  |  |
|  | **2.1** | Dry cough (worse particularly at night) | 1 | 2 | 9 |
|  | **2.2** | Recurrent wheeze | 1 | 2 | 9 |
|  | **2.3** | Dyspnoea / Recurrent difficulty in breathing | 1 | 2 | 9 |
|  | **2.4** | Recurrent chest tightness | 1 | 2 | 9 |
| **3.** | Evidence of reversibility of airflow obstruction after a short acting bronchodilator through: | |  |  |  |
|  | **3.1** | Clinical observation (mainly in children) | 1 | 2 | 9 |
|  | **3.2** | PEF | 1 | 2 | 9 |
|  | **3.3** | Spirometry | 1 | 2 | 9 |
| **4.** | If the diagnosis was not made by you, do you have data from a hospital specialist that validates it? | | 1 | 2 | 9 |

***Thank you for your assistance!***
