# Supplementary material for: Asthma in an Urban Population in Portugal: A prevalence study
Source: BMC Public Health. 2011 May 19;11:347. doi: 10.1186/1471-2458-11-347 (PMC3121634; doi:10.1186/1471-2458-11-347)
Supplement: Additional file 3 — Appendix 3: Patient Questionnaire [file 1471-2458-11-347-S3.DOC]

**Appendix 3: Patient questionnaire**

## Asthma prevalence study in Matosinhos Health Centre

| **Physician** | |_|_|_|_| (code) | **Date** (day/month/year) | __/__/_____ |
| --- | --- | --- | --- |

## Patient’s data

| **Age** | |_|_| years | **Gender |M|F|** | **Code** | ____________ |
| --- | --- | --- | --- | --- |

## Symptoms

| **1.** | Have you ever had wheezing? | Yes | No | I ignore |
| --- | --- | --- | --- | --- |

If you answered no, please move to question number 6

| **2.** | Have you had wheezing in the last 12 months? | Yes | No | I ignore |
| --- | --- | --- | --- | --- |

If you answered no, please move to question number 6

| **3.** | How many attacks of wheezing have you had in the last 12 months? | | | | | | | |  |
| --- | --- | --- | --- | --- | --- | --- | --- | --- | --- |
|  | None |_| 1 to 3 |_| 4 to 12 |_| More than 12 |_| | |  | |  | |  | |  |
| **4.** | | In the last 12 months have you had wheezing during or after exercising? | | Yes | | No | | I ignore | |
| **5.** | | In the last 12 months did you ever wake up because of your wheezing? | | Yes | | No | | I ignore | |
| **6.** | | In the last 12 months have you had dry cough at night, except for cough associated with an upper respiratory tract infection (common cold)? | | Yes | | No | | I ignore | |
| **7.** | | Have you ever had asthma? | | Yes | | No | | I ignore | |
| **8.** | | Have you ever been told by a doctor that you had asthma? | | Yes | | No | | I ignore | |

## Medication

| **9.** | In the last 12 months did you ever use a blue inhaler (Bricanyl or Ventilan) for your shortness of breath or asthma? | Yes | No | I ignore |
| --- | --- | --- | --- | --- |
| **10.** | In the last 12 months did you ever use a green inhaler (Oxis, Formoterol, Serevent, Dilamax ou Ultra-Beta) for your shortness of breath or asthma? | Yes | No | I ignore |
| **11.** | In the last 12 months did you ever use a brown / orange inhaler (Pulmicort, Flixotaide, Brisovent, Asmo-Lavi ou Budesonide) for your shortness of breath or asthma? | Yes | No | I ignore |
| **12.** | In the last 12 months did you ever use a violet / purple / red inhaler (Symbicort, Assieme, Seretaide, Brisomax, Veraspir ou Maizar) for your shortness of breath or asthma? | Yes | No | I ignore |
| **13.** | In the last 12 months did you ever use tablets (Singulair, Lukair ou Accolate) for your shortness of breath or asthma? | Yes | No | I ignore |

***Thank you for your assistance!***
